# Class Address

**Published:** 1879-04

**Authors:** Frank B. Perry

**Affiliations:** VIRGINIA.


### ARTICLE IV.

### *Class Address.*

BY FRANK B. PERRY, OF VIRGINIA.

Delivered at the 39th Annual Commencement of the Baltimore College of Dental Surgery, March 6th 1879.

To say that I am gratified at this mark of appreciation by my class-mates, in selecting me as their speaker, to deliver an address on such an occasion as this, is both natural and true. Hence the memory of this hour will ever linger amongst the brightest recollections of my college days. But however pleasant and natural it may be for one to surrender himself amid such scenes as these to delightful fantasies of fond association and sentiment; yet the profound responsibility which a kindness and partiality, perhaps too indulgent and generous, has imposed upon me, suggests that the moment has its *duties* as well as its pleasures, and that its opportunity must not be permitted to pass away unimproved.

In all the vast range of topics which literature and the arts and sciences, philosophy, religion and politics can present, what subject can be more appropriate and suitable to this occasion or more applicable to us all, whether as students at college or busy gleaners in the field of life, than that suggested by our *hope* and inspiration and the achievements of man in every age and country. For these have been the themes of poets and orators from the time when Homer sang of gods and Heroes, who combatted for love and honor, down through age and every change and phase of civilization, and has ever proved the *inspiration* that has awakened the human mind and stirred the hearts of man to glorious deeds and deathless fame.

These themes, though old, are like the ancient monuments of Egypt, which still remain the examination and admiration and wonder of the world, and whose secrets have been oft discussed, and whose thrilling story has been thrice told, and of which we never tire nor grow weary. They still excite and interest, and still maintain their grandeur and renown. And as we never tire nor grow weary of listening to the oft repeated story of Greek and Roman triumphs, and of perusing the interesting history of their greatness, splendor and power, so we like to hear our favorite topic discussed and dwelt upon. Indeed no subject is calculated to excite so much interest in us as our own *hopes* and aspirations which are founded to so great an extent upon the achievements of others.

And as in the language of Patrick Henry, we have no light to guide our feet but the light of experience, and no way of judging of the future but by the past, so all experience and observation teach us that our success in life depends upon the devotion of ourselves to a single *profession, object or purpose.* For the range of knowledge has become so wide and extended that to become proficient in all things has become an impossibility, if indeed it ever was possible. He whose thoughtful consideration of man and his characteristics has made his " Essay " famous, and secured the proud honor which are so justly his—has said:

> "Our science only will *one* genius fit,
> So wide is art; so narrow human wit."

How many noble youths of well trained and highly cultured minds, good hearts and noble aspirations, have passed through life ever changing from one thing to another, wasting their talents upon a multiplicity of pursuits and never rising above the common class of mankind.

How many of the most brilliant *star*s of our colleges and universities soon sink into obscurity and disappear among the common class of humanity, owing to a want of energy and *singleness of purpose.* Men, who, if they had have arrived at something *great* and definite, might have risen to distinction and honor in some single pursuit or profession, or have "swayed the rod of Empire." Therefore if we waste our talents and energy in vainly trying every new scheme that is suggested, or in following every year's factions that crosses our path, we can not expect to succeed in any of the pursuits of life.

History and biography tell us upon almost every page of the fatal consequence of such a *short-sighted*, *fickle* and senseless policy.

Coleridge, who possessed a mind second to none of his cotemporaries, died without leaving a complete production of his wonderful talents; it is said that he left over 400 treatise on Metaphysics and divinity but not one of them completed. Dr. Quincy Chatterton and Chisolm with all their talents, never left one elaborate and complete work worthy of their rare gifts and fine powers.

And so with thousands of others to whom reference might be made if time would permit; these however will suffice to illustrate the principles for which I contend.

But do not imagine that for the purpose of pursuing our object, all other knowledge should be neglected or forgotten. For it is only claimed, that you shall make all others subordinate to your one great object, "the aim of life." Do not despise other attainments, but let imminent success in the chosen profession in pursuit be your sole *end*, *aim* and *object.*

Why should we claim inability or imperfection when history and biography every where tells that many apparent impediments and obstacles may be overcome or surmounted. Of those who thus conquered nature herself, was Demosthenes, whose standard of greatness was unmeasurable—infinite. His *aim* was immortality. To reach this he overcame physical disabilities, though most discouraging and harrassing. No task was so difficult, no enterprise so arduous but that his aspiring genius found a way to overcome it, and now

> " His name, a great example stands to show,
> How strangely high endeavors may be blest."

Cicero struggled to be equal to his distinguished teacher, and after infinite *labor and toil he too* achieved his triumph over difficulties and adversity, and won a deathless name.

Napoleon Bonaparte, who in the language of Philip, the great Irish Orator, was a stranger by birth and a scholar by charity, with no friend but his sword and no fortune but his talents, rushed into the line where *rank*, fortune and genius had arrayed themselves, and competition fled as from the glance of destiny.

From a poor orphan of foreign birth, by the power of his talents, his indomitable energy and perseverance, and unconquerable determination, he became the greatest general and monarch that the world probably ever saw or knew.

And *why is it* thus? With few exceptions the eminent and distinguished orators, statesmen, warriors, professional men, artisans, inventors, and discoverers in every age and country, sprang from the comparatively humble walks of life; often indeed from the very poorest class of beings. Simply because the labor and application incident and necessary to success is so great that the most wealthy and favorite of our race are not willing to begin at the lower round of the ladder and endure the trial and privation absolutely necessary to reach the top. Hence the lower ranks of every profession are crowded and clogged. But, my young friends and class-mates, there is always an abundance of room at the top. The upper ranks are never crowded ; the room there is ample and the rich reward of success so great as to elicit all our efforts, excite all our energies and inspire us with the most laudable ambition. And it is a matter of especial gratification that our lot has been cast in the grandest age and country the world ever saw. The war cloud that once hung so remorsely and destructively over us has happily passed away and heavenly peace now, spreads her golden wings over us from the Lakes to the Gulf, and from Ocean to Ocean, diffusing her rich blessings all over our happy, *glorious* native land. Sectional animosities are dying away, and we are brother again from Maine to Georgia and the far off Pacific shores, where our rivers roll down their golden sands. Our harvests are bounteous, our currency unsurpassed, our commerce whitens every sea of the known world, our manufactures are without a successful rival, and prosperity is fast returning and blessing every portion of our great and grand republic. Hence wealth and peace and sparkling beauty stand invitingly before us. And therefore if we do not win it will be our fault, with no one to blame but ourselves. We have here contracted a friendship and intimacy which has bound us together, that to the latest day of our lives we will recall,

> "For there is a skill that binds the past,
> Which naught but death alone can sever;
> And memory with retentive grasp,
> Doth call it to the mind forever."

*Gentlemen of the Faculty.*—We acknowledge your ability, and your constant readiness and desire to instruct us. Never can we forget the kindness and forbearance which you ever manifested towards us in the whole course of our difficult and arduous studies. Therefore it affords us infinite pleasure to express to you our appreciation of your efforts in our behalf, and our gratitude for your watchful care, your invaluable instruction, your constant efforts to infuse into our minds the science and mysteries of Dental Surgery, and impress us with the importance of our duties and point out the road to success in this *noble* and humane profession.

To you citizens of Baltimore, we extend our grateful acknowledgements for the kindness and courtesy which so many of you have shown us, during our brief sojourn in your beautiful and flourishing city—thus maintaining your high reputation for those noble virtues, hospitality, affability and good will, and leaving impressions upon our minds that time will never obliterate, and exciting sentiments of friendship in our hearts that will ever cause us to rejoice in your welfare and happiness and the progress of your city in wealth and commerce.

May the star of her destiny never grow dim, her wealth and beauty increase year by year, and her commerce extend until the sails of her ships whiten every sea of the known world, and her railroads penetrate the remotest regions of the great continent.

